# Quantifying Biofield Therapy through Biophoton Emission in a Cellular Model

**DOI:** 10.31275/20201691

**Published:** 2020-09-15

**Authors:** Jeremy B. Kent, Li Jin, Xudong Joshua Li

**Affiliations:** Department of Family Medicine, University of Virginia Athletics, University of Virginia Health System, Charlottesville, Virginia USA; Department of Orthopaedic Surgery, University of Virginia; Department of Orthopaedic Surgery, University of Virginia Health System

## Abstract

Biofield therapy has shown positive results over a broad range of pathology from preclinical research to human studies. However, biofield therapy investigation is limited by an inability to quantify the therapeutic effect. This study aimed to measure the effects Reiki had on mice intervertebral disc (IVD) cells compared with sham and to quantify Reiki by measuring photon emission. We treated mice IVD cells with ten-minute sessions of either Reiki or sham on three successive days. During treatment, we placed the cells in a specifically constructed box with an installed photomultiplier tube (PMT). Reiki significantly increased the photon emission of the cells post-treatment compared with Reiki pre-treatment and sham (*p* < 0.05). Real time PCR (RT PCR) showed an increase in collagen II and aggrecan (*p* < 0.05). We present a means to quantify biofield therapy by measuring the post-treatment photon emission. We concurrently demonstrate Reiki’s effect on the anabolic healing response.

## INTRODUCTION

Biofield research is an emerging area of study (Muehsam et al., 2015; Rubik et al., 2015). The biofield is a complex organization of subtle energetic forces that maintains and regulates the delicate balance of an organism (Rubik et al., 2015). The current agreed-upon theory of the biofield is that it interacts directly with the organism and external environment and may be as much a conduit for information transmission as it is for energy transfer (Rubik et al., 2015). Biofield therapy seemingly works within this framework to manipulate the biofield to positively affect illness (Rubik, 2002).

Biofield therapy has shown positive results over a range of ailments, including the reduction of pain and stress (Anderson & Taylor, 2012; Hammerschlag et al., 2014; Jain & Mills, 2010). Preclinical research in Therapeutic Touch decreased the immune response in mice injected with breast cancer cells and increased osteoblast DNA synthesis and mineralization of human osteoblasts (Gronowicz et al., 2015; Jhaveri et al., 2008).

The mechanism through which biofield therapy acts is unknown. Research has investigated different modalities to describe biofield therapy such as the electromagnetic field properties of electricity and magnetism, sound, and pH (Joines et al., 2012; Kokubo et al., 2007; Kokubo & Yamamoto, 2007; Matos et al., 2017; Muehsam et al., 2015; Rubik & Jabs, 2017). While these different options hold promise, the impasse that continues to deadlock biofield therapy research is the combination of an unclear mechanism and the inability to quantify the dose or the therapeutic effect.

Previous research has based the therapeutic dose on session time (Gronowicz et al., 2015, 2016; Jhaveri et al., 2008). However, session time is likely an inaccurate means to measure the strength of biofield therapy. Biofield practitioners have different training levels and experience. Some people may be more adept than others in promoting healing. As a result, questions remain about the reliability of time as a factor in testing. Consequently, if a study is unable to demonstrate the reliability of the practitioner, the fidelity of the results comes into question.

A solution may lie in the potential link between biofield therapy and the light emitted from living organisms, called biophoton emission (BE) (Ives et al., 2014). BE, also named ultra-weak photon emission, autoluminescence, and spontaneous chemiluminescence, is the discharge of very small amounts of light measured in photons and seen in all living organisms (Cifra & Pospíšil, 2014; Popp et al., 1984) BE has been postulated as a mechanism to further investigate biofield therapy (Ives et al., 2014; Kafatos et al., 2015; Muehsam et al., 2015; R. Van Wijk et al., 2014).

BE research began in the early twentieth century with the discovery that certain cells communicated by emitting small amounts of light in the ultraviolet (UV) to visible spectrum (Cifra et al., 2011; Cifra & Pospíšil, 2014). One hundred years later the details of this communication are still unclear as it is uncertain how cells spontaneously produce light and through which receptors the light is translated to stimulate change. There are a number of different theories on how this occurs, including production of photons within DNA, within mitochondria, at the cell membrane, or through the cell cytoskeleton (Cifra et al., 2011; Dotta et al., 2011; Jibu et al., 1994; Kumar et al., 2016; Prasad et al., 2014).

BE research now encompasses several different subdisciplines to include cell-to-cell communication, organism pathology, organism health, and biofield research (Cifra et al., 2011; Cifra & Pospíšil, 2014; Ives et al., 2014; Kafatos et al., 2015; Kucera & Cifra, 2013). BE is defined as photon radiation generated from the cell’s endogenous energy storage. BE research can be further divided into BE that is spontaneously generated and BE that is induced. Induced BE is typically caused by stress on the organism or cell by factors such as infection, mechanical stress, and ionizing radiation (Cifra & Pospíšil, 2014).

Elevated oxidation and free radical production are well-documented processes associated with disease states (R. Van Wijk et al., 2014). When researchers established links among oxidation, free radical production, and an increase in BE, this led to further investigation of using BE as a means to measure an individual’s health (E. P. Wijk & Wijk, 2005; R. Van Wijk, 2001). Recent studies have shown an association of increased BE in multiple sclerosis patients (Hammann et al., 1987), ankylosing spondylitis (K. J. Ho et al., 2000), and in chronic lung disease (Koval’chuk et al., 1998). This increase in BE during stress, illness, and disease (Grasso et al., 1992; Keshavarzian et al., 1992; E. P. Van Wijk et al., 2010) corresponds to a number of studies that show that BE decreases in healthful behavior such as meditation (E. P. Van Wijk et al., 2006; E. P. Van Wijk et al. 2008a; E. P. Van Wijk et al., 2008b).

In apparent contrast, a few studies have demonstrated an increase in BE after performing biofield therapy (Joines et al., 2012; Kokubo et al., 2007; Rubik & Jabs, 2017). Joines et al. studied BE in more than 100 persons with the intent to perform biofield therapy. They noted a significant increase in the quantity of photons measured in the ultraviolet spectrum when comparing the BE of a biofield practitioner during biofield therapy with the BE of a non-biofield practitioner (Joines et al., 2012). Rubik et al. (2006) noted a significant increase in BE pre-treatment compared with post-treatment, measured from the practitioner’s palms. They also noted a trend that a biofield practitioner’s intention to increase BE did just that (Rubik & Jabs, 2017).

It is unclear why a discrepancy exists between BE studies on meditation and biofield therapy. Presumably, meditation and biofield therapy would have similar BE results. The disparity may arise from the difference in the intent of the participants. Biofield practitioners set their intent to manipulate someone or something else, while persons in meditation set their focus inward on self-reflection.

Other research has investigated biofield therapy’s effect on the subjects that it is directed toward. Kokubo et al. measured the BE of cut cucumbers after “laying-on-hands” and found an increase in BE compared with controls measured post-treatment (Kokubo et al., 2007; Kokubo & Yamamoto, 2007).

An important distinction in BE research is that of delayed luminescence. While BE is the production of photons from the cell’s own energy supply, delayed luminescence is the emission of photons induced by an external light source. A light source stimulates the cell to emit photons through the principles of quantum optics and the photoelectric effect as well as through the photochemical cascade at the cellular level (Hüfner, 1996; Lakowicz, 2006; Martin & Wiese, 2006). Delayed luminescence has typically described a process in plants with an established “photosystem,” but research has shown that other organisms also produce delayed luminescence (Scordino et al., 2014).

The differentiation between BE and delayed luminescence is significant when investigating biofield therapy, since each entails different mechanisms of action. If the mode of biofield therapy is BE, as evidenced in previous studies (Joines et al., 2012; Rubik & Jabs, 2017), then biofield therapy would be a light source. Biofield therapy as a light source would stimulate photon emission through delayed luminescence. As an example, the study results reported by Kokubo et al. (2007) would be a form of delayed luminescence.

Conversely, if the mechanism through which biofield therapy produces effect is not light, then the photons emitted from the subject is BE. The subject is stimulated to generate photons without a light source and instead through an endogenous energy pathway. Differentiating the effects biofield therapy triggers in a subject, whether it is BE or delayed luminescence, is evidence for biofield therapy’s mechanism. In this paper, we use three terms to describe the emission of photons, including biophoton emission and delayed luminescence as described above, and photon emission. We use the term “photon emission” to describe the radiation of photons without discriminating between the mechanism. We use “photon emission” primarily when it is unclear which mechanism is being demonstrated, BE or delayed luminescence.

The biofield therapy this study utilized was Reiki. Reiki purportedly channels the universal life energy to enhance healing. Reiki uses the practitioner’s hands as an important connection with the subject. Reiki students learn through in-person seminars and workshops, graduating from first degree to second degree to the master level over the course of years of practice (Rand, 2000; Stein, 1995). Reiki is an apt technique for the study’s design that includes the practitioner placing their hands in a box. Reiki has been studied extensively for treating pain, anxiety, depression, and in vitro bacterial cultures (Joyce & Herbison, 2015; Rubik et al., 2006; Thrane & Cohen, 2014). In this paper, we use the term “Reiki” to refer to the practice itself and not to the reiki energy or life force that Reiki channels. We use the term biofield therapy as a broader description of a number of similar techniques including Reiki, which other published articles have described in equivalent terms (Rubik et al., 2015).

To test the effects of Reiki, we used mouse intervertebral disc cells (IVD). IVDs consist of a fibrocartilage structure made of collagen. Degeneration and damage to the fibrocartilage leads to low back pain (Huang et al., 2018). Discogenic pain is associated with low back pain. IVD cells are well-established in the study of low back pathology (Liu et al., 2013). Research has demonstrated that collagen tissue displays delayed luminescence when stimulated with a light source (M. W. Ho et al., 2002). As such, collagen is an adequate cellular model to investigate BE.

Treatment for degenerative IVD is limited (Casazza, 2012). Recovery can be prolonged and chronic pain is not uncommon. Biofield therapy has the potential to improve pain and augment the healing process.

In this paper, we report on investigations of biofield therapy through measuring photon emission. We conducted our research using a custom light-tight box with an installed photomultiplier tube (PMT) described below. We made two assumptions based on previous data as detailed above:

1. Biofield therapy practitioners with the intent to perform biofield therapy increase BE from their hands.
2. Stressed IVD cells stimulate an increase in BE.

We hypothesized that Reiki directed at IVD cells stimulates an increase in photon emission post-treatment. We concurrently examined the effectiveness of Reiki by measuring anabolic extracellular matrix synthesis markers of IVD cells.

## METHODS

### Light Box Construction

We constructed two boxes out of plywood to create a very-low-light environment, which ensured accurate measurement of the small amount of light released in BE. The box’s dimensions were 710 × 510 × 510 mm and 790 × 560 × 580 mm. We coated the box edges with black sealant. The smaller box fit into the larger box. A nonreflective black polyester fabric covered the interior of the smaller box. Reflectix reflective roll insulation (Reflectix, Markleville, IN) was wrapped around the outer box. We cut two 130-mm-diameter holes on one side of each box for hand placement. We stitched two sleeves, each three feet long and made of a cotton and spandex blend (90%, 9%, respectively). We covered each sleeve in Reflectix and attached one sleeve to each cutout. On the lid of the inner box was mounted a polystyrene foam box with a PMT, described below, installed inside. On the floor of the box, we placed an elevated wooden tray that covered the width of the box. We made a 153-mm-diameter circular cutout in the middle of the tray. We threaded a nylon wire mesh over the tray cutout to act as a platform to hold the cell plates. An aluminum conical connected the tray to the PMT.

**Figure 1.**
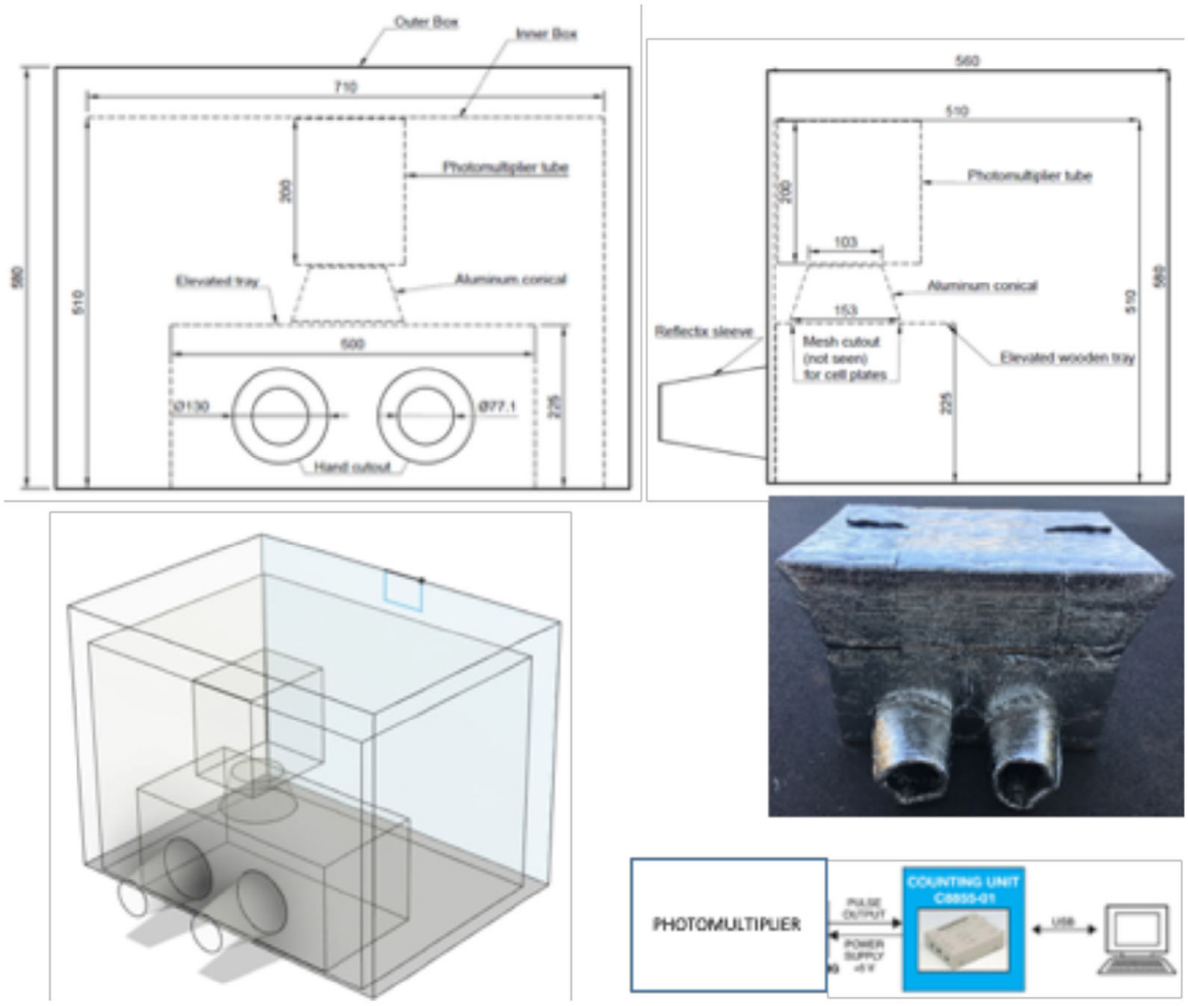
Design of the light box that was tailored to measure biofield therapy from the hands of practitioners. The practitioner placed their hands through the cutouts, within the elevated wooden tray and underneath the cell plate tray. The cells lay on the cell plate tray. The PMT box houses the PMT (not shown); the cables thread through the upper window flaps. The schematic details the connection of the PMT via the counting unit to the laptop PC. All measurements are in millimeters.

The conical encased the cell plates lying on the mesh and aligned them directly under the PMT separated by a distance of 76 mm. Figure 1 is a photograph and a schematic of the light box. The practitioner placed their hands through the sleeve and the two box cutouts, positioning their hands under the nylon mesh of the tray, palms up, facing the cells. We conducted baseline readings of the light box in a room with a low level of light. The baseline readings were 6.8 ± 3.0 photons per second (CPS). The readings were equal to the manufacturer’s advertised dark count for the PMT used in this study.

### Biophoton Emission Measurement

We used a PMT (Hamamatsu model H6240-1 side-on photomultiplier module, Hamamatsu Photonics, Japan) with an effective spectral response range of 185–850 nm. The photocathode window measured 8 mm by 24 mm. We attached to the PMT an infrared cutoff filter, Schott KG-1 heat-absorbing glass 25 mm × 25 mm (Schott, Mainz, Germany) with a transmission of 275–750 nm. The Schott infrared filter was used to block infrared photons. The filter eliminated photon emission as a result of heat. Connected to the PMT was a Hamamatsu C8855-01 photon counter (Hamamatsu Photonics). The photon counter connected to a PC laptop via USB. We used Hamamatsu Control Software for the C8855-01 photon counter (Version 2.00), counting one photon per second. The PMT was cooled to 5 °C and housed in a polystyrene foam box. Ambient temperature of the room where we conducted the study averaged 23.3 °C on day 1, 22.2 °C on day 2, and 22 °C on day 3.

### IVD Cell Isolation

All procedures involving animal materials were approved by the Institutional Animal Care and Use Committee of the authors’ institution. Mice were sacrificed and the lumbar discs were dissected and cultured in a 60-mm dish at 80–90% confluence and in a 37° C warming incubator, as reported previously (Liu et al., 2013). To simulate pathology and induce cell stress, we supplemented the cell medium with a pro inflammatory cytokine, tumor necrosis factor-α (TNF-*α*, 10 ng/mL) 48 hours prior to the first Reiki or sham treatment. Research has shown that TNF-*α* not only stresses cells, but also stimulates BE from the cells in medium (Madl et al., 2017; R. van Wijk et al., 2010).

### Reiki

One Reiki practitioner participated in this study. This person is a master level practitioner who has more than thirty years of active experience in providing and teaching Reiki. The Reiki practitioner performed Reiki according to standard technique (Rand, 2000; Stein, 1995). One sham practitioner also participated in this study. The sham practitioner had no knowledge of biofield therapy and was asked to think of distracting thoughts such as counting backwards.

**Figure 2.**
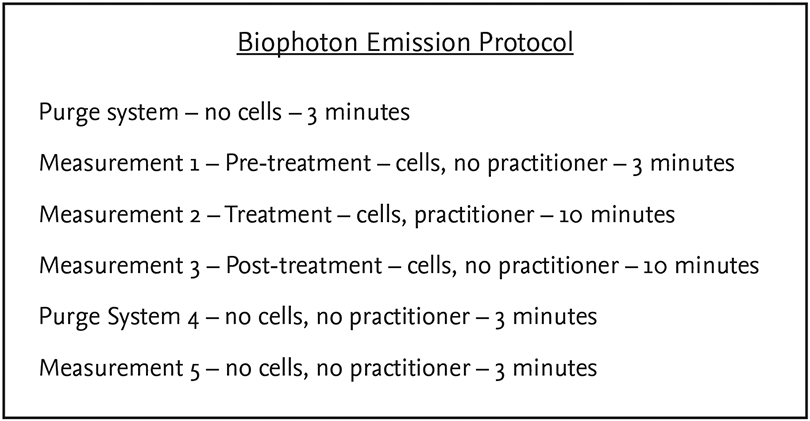
Biophoton emission measuring protocol. Protocol was completed each day for 3 days. Sham was conducted before Reiki.

We conducted the photon emission measurements over a three-day period. At the beginning of each day, we purged the box of light by recording PMT measurements for eight minutes. The protocol to measure the BE is found in Figure 2. Both the Reiki and sham practitioners were exposed to no sunlight at least 20 minutes prior to starting the study to limit them incidentally absorbing light into the skin (E. P. Wijk & Wijk, 2005). We conducted each group in sequence, sham first then Reiki, with minimal delay between groups to switch the practitioner and cells. We designated two cell plates for sham treatment and two cell plates for Reiki treatment. We placed both cell plates in the box on the tray concurrently during treatment. After treatment, a warmer housed the cell plates to be utilized for each treatment day. Treatment lasted 10 minutes. This was duplicated for Reiki and sham treatments for a total of three days. We chose three days based on a previous successful protocol we developed utilizing the same cell and molecular biology techniques (Liu et al., 2013). The study was conducted in February 2017 in a nondescript lab room with no windows in a large research facility.

**Figure 3.**
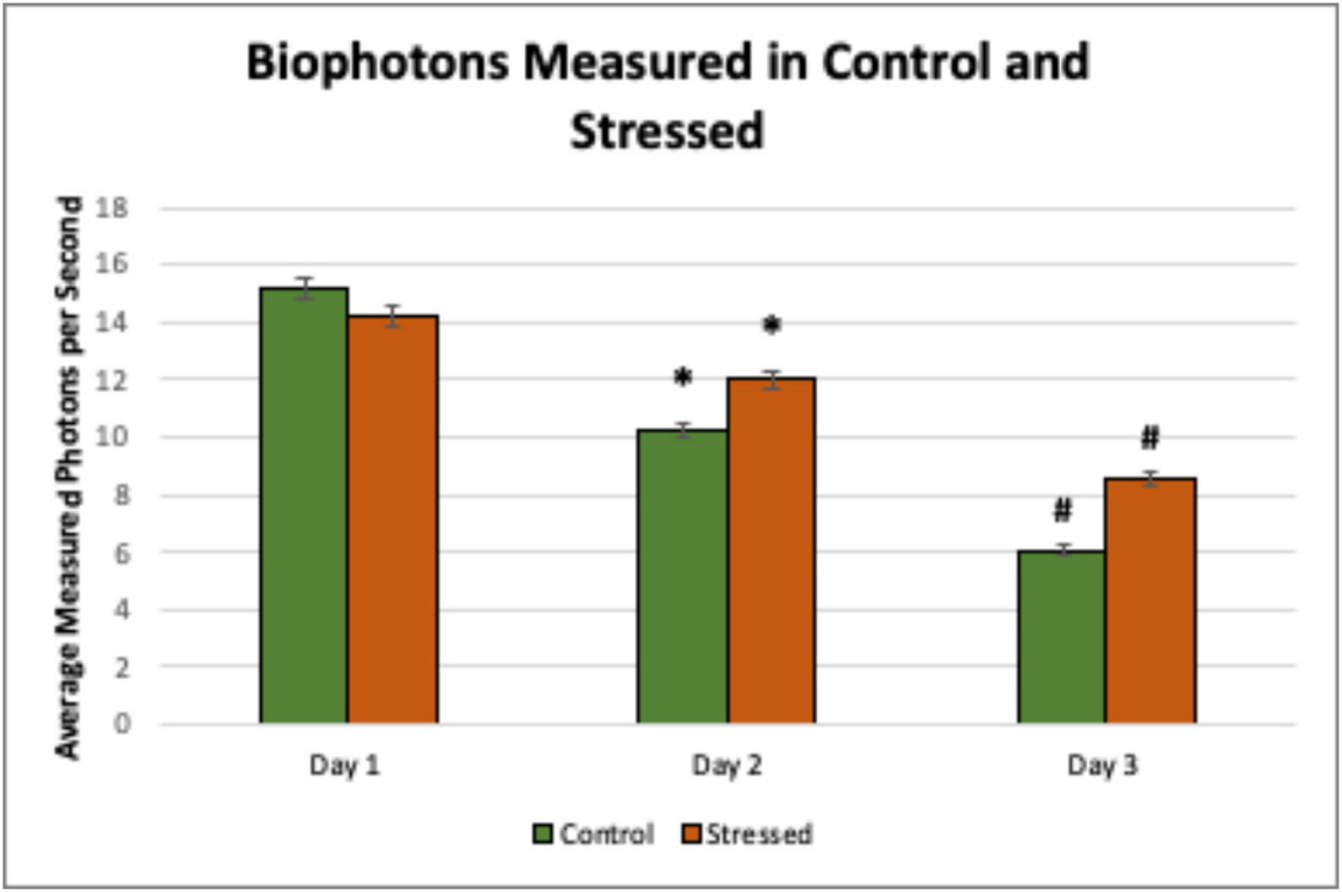
We measured the internal validity of the light box by comparing the BE of stressed intervertebral disc cells with TNF-α to unstressed cells over a three-day period. The results on Days 2 and 3 were as expected. Day 1 was not as expected, but we attribute this to a short time period to allow the stress to produce BE (*p < 0.05, #*p* < 0.05).

We measured the internal validity of the light box by comparing the BE of stressed IVD cells to unstressed cells over a three-day period (Figure 3). Day 1 of the internal validity test was not significant. We theorize that the first day was inconsistent with expected results due to the short time to induce stress. Days 2 and 3 showed a significant increase in BE from the TNF-α supplemented cells compared with the control cells, as expected (Figure 3).

### PCR Assay

Twenty-four hours after the last Reiki treatment, we extracted total RNA from the disc cells using the Reagent QIAGEN (Thermo Fisher Scientific, Waltham, MA). We determined the RNA concentration using a Nanodrop spectrophotometer at 260 nm. Complementary DNA was synthesized using the iScript cDNA Synthesis Kit (Bio-Rad, Hercules, CA). Quantitative PCR containing SYBR green master mix Real-time PCR was performed with iQ 5 multicolor real-time PCR Detection System (Bio-Rad, Hercules, CA). The mRNA expression of anabolic genes collagen I, collagen II, and aggrecan was evaluated. We used 18S rRNA as an internal control.

### Statistics

We performed all experiments with duplicate cell plates. Statistical analysis for quantitative assays was performed by 1-way analysis of variance assuming equal variance using Microsoft Excel software version 14 (Microsoft, Redmond, WA, USA). A *p* value of less than 0.05 was considered statistically significant.

## RESULTS

### Biophoton Emission

**Figure 4.**
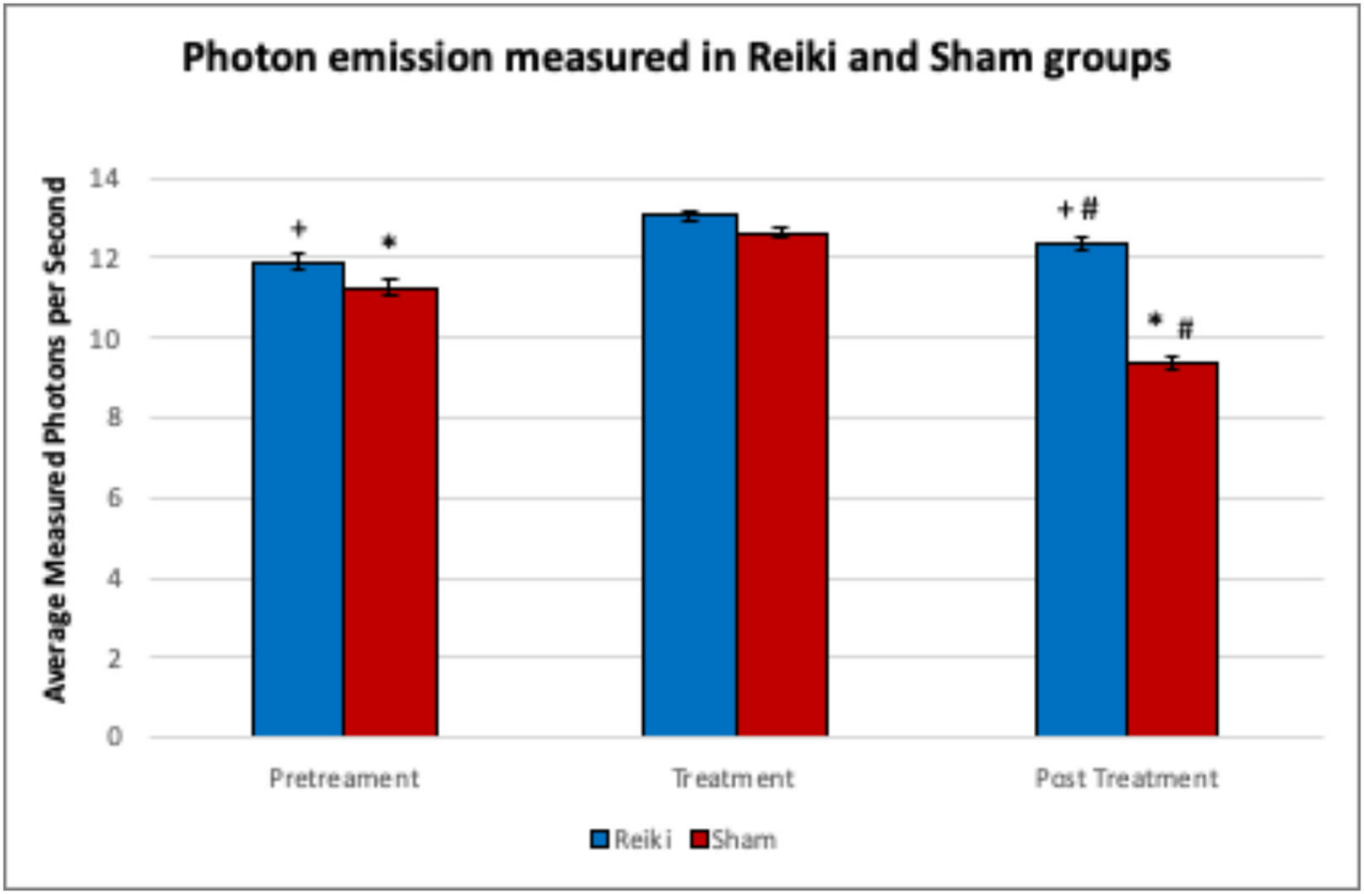
The average number of biophotons emitted per second for Reiki and sham groups over the three treatment days. Post-treatment photon emission was significantly different from both Reiki pre-treatment and Reiki post-treatment. Photon emission was significantly different between Reiki post-treatment and sham post-treatment (+*p* < 0.05, #*p* < 0.05, *P < 0.05).

**Figure 5.**
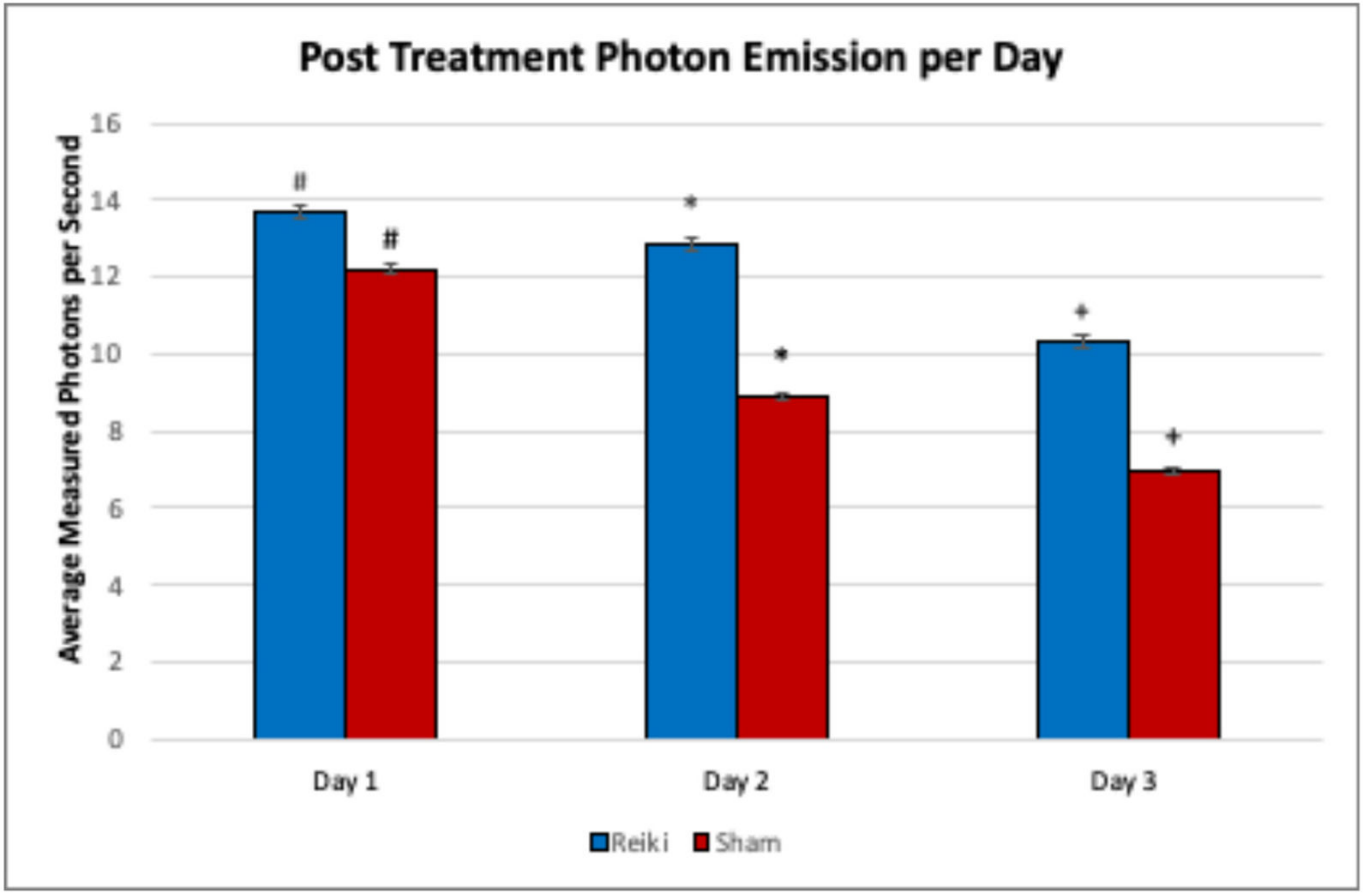
Comparison of photon emission for post-treatment groups of Reiki and sham on days 1, 2, and 3. Reiki increased photon emission compared with sham on each day measured (# *p* < 0.05, *p < 0.05, +*p* < 0.05).

The study found a statistically significant difference between photon emission measured in Reiki post-treatment compared with the Reiki pre-treatment (+*p* < 0.05) (Figure 4). When comparing Reiki and sham groups during the post-treatment period, Reiki showed a significant increase in photon emission compared with sham (#*p* < 0.05) (Figure 4). On a day-by-day comparison, post-treatment Reiki maintained a higher BE than the post-treatment sham group (*p* < 0.01) (Figure 5). We found no difference in BE between Reiki and sham during treatment (Figure 4).

### RT PCR

**Figure 6.**
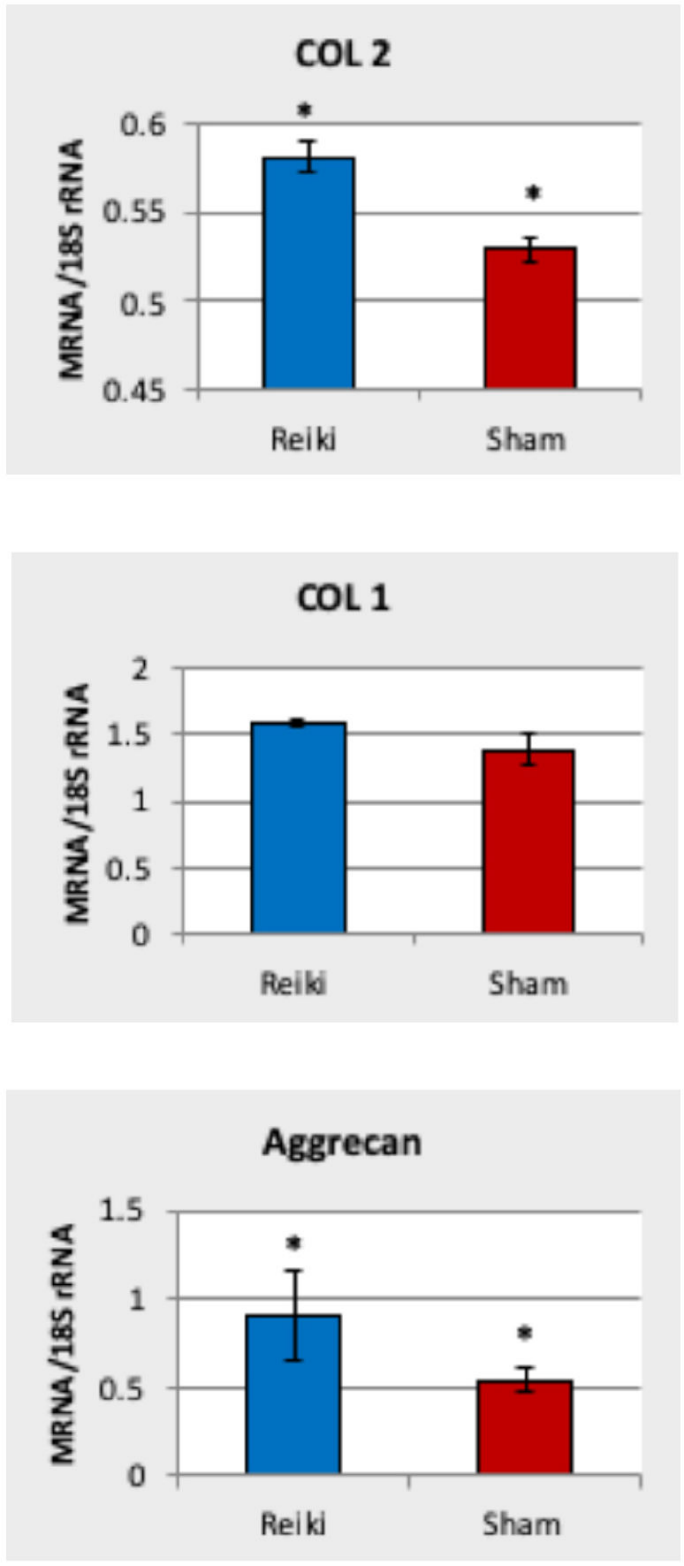
Reiki enhanced intervertebral extracellular matrix gene expression. At day 3, total RNA was extracted and gene expression of COL1, COL2, and aggrecan was evaluated by real-time PCR (*p < 0.05). Reiki showed a significant difference in COL2 and aggrecan from sham. Although increased from sham, gene expression for COL1 was not significant.

Healthy disc cells maintain a balance between anabolism and catabolism. During aging and degeneration, the imbalance may reduce cell viability and extracellular matrix synthesis. Collagen II (COL2), collagen I (COL1), and aggrecan are typical anabolic matrix proteins. Reiki and sham treatments were compared after TNF-α administration. Reiki significantly increased COL2 compared with sham (*p* < 0.05). It also significantly increased aggrecan over sham (*p* < 0.05). Reiki increased COL1 over sham, but this was not significant (Figure 6).

## DISCUSSION

Although the evidence for the positive effects of biofield therapy continues to mount, biofield therapy research remains hampered by our inability to quantify the therapeutic effect. This study investigated BE and delayed luminescence to quantify biofield therapy.

The results showed that post-treatment photon emission from Reiki-treated cells was higher than pre-treatment BE (Figure 3). These results are consistent with Kokubo et al. (2007) who showed an increase in photon emission after “laying on of hands.” Post-treatment photon emission is a measure of the subject’s response to Reiki. Response is a means to indirectly quantify a treatment. To directly quantify a treatment, the treatment composition must be known, in order to calculate a dose-response relationship. Since Reiki’s mechanism and structure have not yet been clearly identified, we must rely on indirect methods to quantify it. Our data support the conclusion that the difference between post-treatment photon emission and pre-treatment BE is a measure of biofield therapy.

This study was novel in two major respects. It combined several different subcategories of photon emission including spontaneous BE, stress-induced BE from TNF-α, and delayed luminescence. The study design also measured photon emission at multiple time points while concurrently measuring the cellular response to biofield therapy after induced pathology by TNF-α. However, the complexity of the study also produced some limitations. Primarily, our study results did not support our initial assumptions of BE, leaving unanswered questions. We presupposed, based on previous research, that the Reiki mechanism is BE (Joines et al., 2012; Kokubo et al., 2007; Kokubo & Yamamoto, 2007; Rubik & Jabs, 2017). Because of this, we expected Reiki BE to increase compared with sham BE during treatment if all other factors were equal including the TNF-α induced stress on the cells. However, Reiki and sham BE were not significantly different in the treatment group. An explanation for the Reiki treatment group measurements being lower than expected is that the Reiki-induced cells absorbed the emitted photons from the practitioner, which prevented the PMT from measuring the photons. Instead, the photons were measured post-treatment according to delayed luminescence. As a result of a lower- than-expected Reiki measurement during treatment, we cannot confirm that BE in the 275–750 nm wavelength range is the mechanism for biofield therapy. Consequently, we cannot determine which form of photon emission occurred, post-treatment BE or post-treatment delayed luminescence. In fact, both forms of photon emission may be occurring concurrently (Cifra & Pospíšil, 2014). We also cannot make conclusions with respect to the discrepancies that exist among studies measuring the BE of diseased subjects, meditation, and biofield practitioners. Two different processes may be occurring. The intent of the subject tested is likely a factor in this relationship, which could be a basis for a different mechanism of action. The state of the subject might also be a factor. A subject that is in a state of equilibrium may not emit as much BE as a subject that is in disequilibrium. If so, then comparing the BE of diseased subjects, meditation, and biofield practitioners as similar mechanisms may be incorrect.

We assumed that BE would increase during biofield therapy treatment and this increased quantity of BE would indicate a greater therapeutic effect. However, the sheer quantity of BE may not be the driving force that enhances the therapeutic effect. The mechanism of action might be through another form of information transfer not related to the bulk photon energy transmission of delayed luminescence. This would correspond with the original discovery of BE as a means of cell-to-cell communication. Other authors have described this as a coherent state of the biophoton field (Popp et al., 1984; Popp et al., 2002). In which case, categorizing all forms of BE as communication may be a more apt description of the process instead of subdividing BE. All BE may be a form of communication to the external environment. Consequently, the absolute quantity of BE is not as important as understanding the patterns within BE. Similar ideas were put forth by Rubik et al. (2015) when they described the biofield in terms of biofield communication and biofield regulation. This line of thought would also coincide with the state of equilibrium of the subject. An organism in a balanced state would not need to communicate with the environment as much as one that is stressed or intending to communicate.

Other factors may play a part in the mechanism of BE and biofield therapy, including a wavelength spectrum not measured in this study, photon spin, and momentum. Alternatively, post-treatment photon emission may be the byproduct of biofield therapy and the mechanism through which it occurred might be an entirely different form of energy.

Our second assumption that sham BE should be consistent did not bear out in our study. We found that sham-treated cells saw a decrease from pre-treatment BE to post-treatment BE. TNF-α should have induced consistent BE from the stress it produced on the cells. A possible explanation for this difference may be that the cells were overstressed, leading to a decline in their overall function with each successive day. This could explain the stepwise decrease in post-treatment photon emission.

We utilized only one Reiki practitioner. Other practitioners or forms of biofield therapy may produce different BE results. Different treatment durations might cause different results, and the subject’s maximum healing potential may peak after a certain length of treatment period.

The cellular results are consistent with previous preclinical research and show that Reiki enhances the healing cascade in cells. Reiki significantly increased COL2 and aggrecan. COL2 and aggrecan are extracellular matrix proteins critical in the structure and function of cartilage. In damaged or diseased cartilage, COL2 and aggrecan decrease. Reiki attenuated their decrease in cells treated with TNF-*α*. Extracellular matrix proteins also have been proposed as potential generators of BE. Their stimulation in this study may be further evidence of a link between the cell cytoskeleton and BE from endogenous pathways (Cifra et al., 2011; Jibu et al., 1994; Prasad et al., 2014). Future research in biofield therapy would investigate varying treatment durations, different practitioners, and diverse cellular markers in the signaling cascade and healing cascade.
